# Translational bottlenecks in blood-based proteomics

**DOI:** 10.1038/s44321-026-00468-8

**Published:** 2026-06-09

**Authors:** Sara Ahadi, Daniel Hornburg, Haris Babačić, Johannes B Müller-Reif, Lee S Cantrell, Fredrik Edfors, Stefanie M Hauck, Eric W Deutsch, Gilbert S Omenn, Philipp E Geyer, Jochen M Schwenk

**Affiliations:** 1 Independent Researcher, San Mateo, CA USA; 2 Bruker Daltonics Inc., Billerica, MA USA; 3 SciLifeLab, Department of Oncology-Pathology, Karolinska Institute, Stockholm, Sweden; 4 Department of Proteomics and Signal Transduction, Max Planck Institute of Biochemistry, Martinsried, Germany; 5 Seer, Inc, Redwood City, CA USA; 6 SciLifeLab, Department of Protein Science, KTH Royal Institute of Technology, Stockholm, Sweden; 7 Metabolomics and Proteomics Core, Helmholtz Center Munich, German Research Center for Environmental Health, Neuherberg, Germany; 8 Institute for Systems Biology, Seattle, WA USA; 9 Departments of Computational Medicine & Bioinformatics, Human Genetics & Genomics, Internal Medicine, and Environmental Health, University of Michigan, Ann Arbor, MI USA; 10 Ions.bio GmbH, Planegg, Germany

**Keywords:** Proteomics

## Abstract

This Comment identifies key bottlenecks limiting the clinical adoption of multi-protein blood biomarkers and argues that progress requires a shift from exploratory profiling to decision-oriented proteome analytics with early cross-sector alignment.

**Figure:**
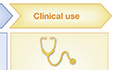

## Teaser

The translation of blood-based proteomics into healthcare is no longer constrained primarily by technological limitations, but by unresolved challenges in standardization, validation, and implementation. In this Commentary, we identify four structural bottlenecks at the current translational inflection point that limit the opportunities for the adoption of multi-protein blood biomarkers in routine clinical care: lack of harmonized reference frameworks, uncertainty around fit-for-purpose biological resolution, complexity in validating multi-analyte and algorithm-based tests, and misalignment between the design of discovery workflows and clinical requirements. We argue that progress will depend on shifting from exploratory profiling toward decision-oriented proteome analytics, with early alignment across academic, regulatory, clinical, and technical domains. Establishing coherent validation pathways for clinically actionable use cases will be essential to enable reliable integration of proteomics into healthcare.

## Introduction

Blood-based proteomics has entered a phase of unprecedented analytical capability and expanded utility outside of the proteomics community. Advances in high-resolution mass spectrometry, affinity-based platforms, and data processing now enable the measurement and analysis of thousands of circulating proteins across large cohorts with increasing precision and scale (Eldjarn et al, 2023; Kirsher et al, 2025). Population-scale initiatives such as the UK Biobank (Koprulu et al, 2026) have demonstrated that proteome-wide profiling can be deployed reproducibly across tens of thousands of deeply phenotyped individuals. At the same time, minimal invasiveness and the ability for longitudinal sampling enable dynamic profiling of serum, plasma, and extracellular vesicles, supporting screening and risk prediction, early disease detection, therapeutic monitoring, patient stratification, and causal inference in drug development (Zhou et al, 2019; Bader et al, 2023). In therapeutic contexts, proteomics offers actionable readouts of efficacy and safety, informs companion diagnostics, and clarifies mechanisms of action. Patient-centric microsampling approaches, including dried blood spots, further enable decentralized, repeatable collection, expanding access to liquid biopsy material and improving inclusion of vulnerable populations (Shen et al, 2024).

Despite these advances, clinical translation has remained limited.

The central barrier is no longer technological capacity, but rather alignment with clinical needs and translational infrastructure. Analytical advances have outpaced the regulatory frameworks, validation standards, computational governance, and clinical integration pathways required for routine implementation. The critical challenge is no longer the discovery of additional biomarkers deep inside the proteome, but establishing the reproducibility, standardization, and decision-anchored design necessary to render multi-protein measurements clinically actionable and useful. Here, we outline four of the current structural bottlenecks that need to be addressed for blood-based proteomics to fulfill its translational promise.

## Standardization and reference frameworks

The challenge begins with the sample. Variability in sample collection, processing, and storage influences analytical workflows, continuing to cause inconsistent outcomes across studies, even when technically robust platforms or harmonized standard operating procedures are used. Differences in pre-analytics, susceptibility of proteins, assay-specific quality control thresholds, and data processing pipelines limit reproducibility and complicate meta-analyses. Without further harmonization of procedures and addition of sample-specific information to analysis plans, cross-cohort validation remains fragile and regulatory confidence difficult to establish. Sparse re-sampling schemes further restrict the ability to detect transient or time-dependent molecular changes.

During the last decade, HUPO’s Human Plasma Proteome Project (HPPP) has taken important steps toward informing about these challenges in a growing technology landscape (Schwenk et al, 2017; Deutsch et al, 2021) by proposing standardized protocols, quality control metrics, and reporting guidelines (Geyer et al, 2024). Broader adoption and enforcement of such harmonized frameworks across large consortia and translational studies would significantly strengthen reproducibility and cross-study comparability (Distler et al, 2025; Kardell et al, 2025).

Even with standardized or kit-based workflows that are becoming more widely used than lab-proprietary procedures, the field lacks universally accepted blood serum or plasma reference standards that enable calibration across the different platforms. Without common reference materials, as tested during early HPPP efforts (Omenn et al, 2005), cross-technology benchmarking remains incomplete, making optimization difficult and limiting integration of proteomic datasets into regulatory pipelines. This limitation is particularly consequential for multi-protein panels, where cumulative analytical variability complicates the use of composite scores. A recent proposal for shared plasma reference materials, described by (Cai et al, 2025), provides a first practical pathway toward cross-platform normalization and benchmarking. By introducing standardized, well-characterized blood samples for calibration and quality control, such frameworks aim to bridge otherwise disparate datasets and enable consistent comparisons across studies, clinical sites, and technologies. Establishing these as technology-agnostic reference materials will be central to improving reproducibility beyond isolated efforts, supporting regulatory-grade validation and integration of multi-analyte proteomic measurements into clinical workflows. Broad implementation of such reference standards should be prioritized as foundational cornerstones for regulatory-grade assay development.

Beyond sample-level standardization, challenges also arise at the level of insufficient resolution and knowledge about proteoforms, partly due to the lack of community standards for reference sequence databases. Proteomic measurements are commonly reported using canonical protein group identifiers that collapse unresolved isoforms and proteoforms into composite protein entities. While this practice is often necessary for both mass spectrometry- and affinity-based workflows and enables robust large-scale analyses, it can obscure biologically distinct molecules, limit functional interpretability, and hinder cross-platform interoperability. This loss of proteoform information can be relevant from a biomedical perspective, when only specific proteoforms of a given protein have clinical predictive value but not others, and from a technological perspective, by reducing assay accuracy and precision and external validity of proteomic findings. Standardized annotation frameworks, including consensus reference databases with comprehensive proteoform inclusion or standardized de novo approaches for variable sequence mapping, could facilitate comparability between studies. Such frameworks may reveal proteoforms with distinct biological and clinical relevance, enhance translation, and improve generalizability across patient populations.

## Biological resolution and fitness-for-purpose

Current proteomic technologies, whether affinity- or mass spectrometry-based, capture a substantial yet inherently incomplete and only partially overlapping view of the circulating proteome. Analytical coverage, defined as the cumulative number of detected analytes across samples, remains far below the ~20,000 protein-coding genes, and it further shrinks when searching for proteins consistently detected in every sample. Thus, the proteomic target list is orders of magnitude away from the millions of potential proteoforms arising from genetic variation, alternative splicing, and post-translational modifications (Aebersold et al, 2018).

Despite this, increasing proteome depth alone does not ensure clinical utility. Expanding molecular coverage often introduces additional sources of uncertainty, including increased analytical variability (Korff et al, 2025; Gao et al, 2026) and limited orthogonal validation options to address specificity and cross-reactivity (Kirsher et al, 2025; Benson et al, 2019). In addition, highly multiplexed assays have a limited capacity for finding the optimal conditions for every analyte, causing a compromise that may compress the dynamic range and resolution. Affinity-based platforms, for example, have faced specificity constraints even though they provide a broad, pre-defined list of target panels measured at pre-defined sample dilutions. In contrast, mass spectrometry-based approaches offer inherent molecular flexibility, as the technology seeks to measure all detectable components, but remain constrained by sensitivity, dynamic range, coupled with trade-offs in throughput and standardization. At present, neither approach alone can comprehensively resolve the full spectrum of clinically relevant proteoforms at scale. Consequently, data from both approaches are often used in a complementary manner (Kirsher et al, 2025).

The central challenge is therefore not maximal coverage, but appropriate and biologically meaningful resolution for the intended clinical application. In some settings, aggregate protein-level measurements are sufficient and may offer greater robustness, as exemplified by widely used clinical immunoassays targeting specific epitopes. In other settings, however, proteoform-level and interference-free resolution may be essential because different molecular forms of the same protein can carry distinct biological and clinical information. For example, distinguishing apolipoprotein E proteoforms is relevant for Alzheimer’s disease risk assessment because the isoform state, rather than total ApoE abundance alone is linked to risk profiles (Weber et al, 2024). Similarly, specific glycosylation states of apolipoprotein C-III have been associated with vascular complications in type 2 diabetes (Naber et al, 2024), illustrating that post-translational modification patterns can encode disease-relevant information that would be obscured by reporting only total protein abundance. More broadly, isoform- or modification-specific measurements may be required when the clinical question involves pathway activation, proteolytic processing, immune activation, or drug mechanism of action. In such cases, collapsing measurements to a single protein-level value may mask the relevant biology, weaken biomarker performance, or complicate therapeutic monitoring. Recent advances in Alzheimer’s disease biomarkers further highlight the importance of proteoform-aware measurements, as phosphorylation-specific plasma tau assays (e.g., p-tau231 and p-tau217) outperform total protein measurements in detecting early amyloid pathology and are moving blood-based diagnostics closer to clinical application (Milà-Alomà et al, 2022).

Determining which level of resolution is required must therefore be guided by the clinical question, not by technological capability. The relevant question is not whether a platform can measure more molecular species, but whether resolving those species changes interpretation, improves classification, supports treatment selection, or provides a more reliable readout of disease biology.

In addition, when considering proteomics more broadly, global proteomics offers the potential to capture multiple disease associations within a single measurement, enabling system-level assessment beyond individual biomarkers. However, its clinical adoption remains limited by challenges in reproducibility, interpretation, and overlapping associations across conditions, which complicate its use in defined decision frameworks. As a result, for well-defined diagnostic applications, targeted proteomic assays will likely dominate the clinical standard as these provide answers to the association in question.

## Analytical validation and regulatory pathways for multi-analyte signatures

Multi-analyte panels that measure several pre-defined proteins at once have received growing attention in the translational field. Large cohort studies have demonstrated that multi-protein risk scores can outperform conventional clinical predictors for cardiovascular and metabolic outcomes (Carrasco-Zanini et al, 2024), yet their validation introduces challenges that extend well beyond traditional single-analyte paradigms. A central issue is defining the regulated entity. In single-analyte assays, validation focuses on analytical performance (e.g., precision, sensitivity, specificity) at clinically relevant decision thresholds for one analyte. In contrast, multiplex proteomic tests generate a composite score derived from multiple measurements, frequently via a fixed algorithm. Regulatory agencies may therefore consider not only each individual analyte, but also the algorithm itself as a medical device requiring independent validation. This raises a fundamental question: is the product the measurement, the model, or the combination of both? Also, can the algorithm be tuned so that the same analytical data will inform about other health conditions? What happens if not all analytes in a multi-marker panel are detected? Will the test still provide clinically informative results? Frameworks to handle those questions have been proposed but will require a coordinated effort across disciplines to introduce in practice (Müller-Reif et al, 2026).

A second challenge lies in analytical and pre-analytical variability, which is amplified by the susceptibility of each analyte in multiplex settings for the samples and calibrators. Inter-laboratory studies have shown that pre-analytical factors, such as sample collection, processing, handling, and storage, can be the dominant source of variability in proteomics assays. For multi-analyte panels, such variability propagates across analytes, as these may not share one common susceptibility, thereby destabilizing the composite score. Regulatory expectations therefore extend beyond standard analytical validation (precision, linearity, limit of detection and specificity) to include rigorous control of pre-analytical workflows, matrix effects, and stability of calibrators and controls. Critically, assay performance must be demonstrated at clinically relevant decision thresholds, where even small deviations can alter classification outcomes.

Third, algorithm definition and validation introduce unique regulatory constraints. When multiple analytes are combined into a single score, the algorithm must be pre-specified (“locked”) prior to clinical validation, and training and validation datasets must be strictly separated to avoid bias. The clinical cutoff or decision rule must be justified using independent data, and performance must be evaluated in the context of the intended use, which ultimately defines the regulatory requirements and acceptable risk profile of the test. Importantly, once validated, modifications to the algorithm, such as recalibration for new populations, may trigger the need for re-validation or regulatory re-review, creating tension with machine learning-driven biomarker development, where iterative updates are common. The development of such version-based and locally fitted algorithm models will require not only new tight regulatory frameworks and validation but also reproducibility, feasibility, and concrete clinical application to facilitate a wider clinical implementation. The fast pace of development of both proteomic methodologies and artificial intelligence methods may require adaptation of existing regulatory frameworks to enable the timely deployment of updated versions of already approved products to the patient.

These challenges have been successfully navigated in genomics, providing a useful blueprint for translational proteomics. Multi-analyte diagnostics such as Oncotype DX (Sparano et al, 2018) achieved clinical adoption by clearly defining the intended use, locking the algorithm, and validating performance in prospective clinical studies tied to specific treatment decisions. This precedent underscores that clinical utility, not just predictive performance, determines regulatory success.

As artificial intelligence increasingly drives biomarker discovery, regulatory agencies are actively defining frameworks for software- and model-based diagnostics (Babic et al, 2025; Singh et al, 2025). Proteomic signatures derived from such approaches will fall within these evolving governance structures, requiring a clear definition of what is being validated, whether individual assay components, the composite output, or the locked algorithm, alongside lifecycle management strategies addressing model updates, performance drift, and transferability across different populations.

The key requirement for clinical translation is therefore not simplification, but specification: defining the regulated entity, controlling sources of variability, and aligning validation strategies with the intended clinical use. Without this, even high-performing multi-analyte signatures will struggle to achieve regulatory approval and clinical adoption.

## Bridging discovery and clinical implementation

Despite rapid technological progress, broader clinical adoption of blood-based proteomics remains constrained by a widening gap between discovery-oriented research workflows and routine clinical practice. Discovery platforms are optimized for breadth, flexibility, and exploratory depth; clinical diagnostics require clear focus, specific implementation, robustness, standardization, rapid turnaround, and regulatory-grade reproducibility, ideally applicable and feasible in different healthcare settings across the globe. These objectives are not inherently aligned.

One manifestation of this gap is limited cross-platform transferability. Proteins identified as predictive on one discovery platform may show incomplete concordance when measured using alternative validation technologies due to differences in molecular readouts (e.g., independent epitope, modified peptides) or the impact of preparative strategies. This also applies to different platform versions (like targeted panel versions) or between different instrument setups following a common preparatory procedure. The currently low cross-platform correlation complicates independent validation and hinders the development of targeted multi-analyte assays suitable for clinical deployment. As a result, promising signatures identified in research settings with one platform may fail upon transfer into stable, clinically deployable formats on a separate platform.

Moreover, multidisciplinary misalignment magnifies the problem. Proteomic studies are often retrospective and prioritize scale, maximizing sample size and proteome coverage, without anchoring assay design to a defined clinical decision point. In contrast, clinical environments require concise, interpretable outputs that directly inform patient management. Clinical decisions are made at an individual, not population, level. Without early engagement of clinicians, regulatory experts, and health economists, technically sophisticated assays risk remaining misaligned with real-world needs. Importantly, clinical proteomics translation will require demonstration of utility, feasibility, safety, efficacy, and cost-effectiveness of a potential blood protein biomarker through prospective clinical studies. Toward this goal, the proteomics community could realign priorities for method development along the clinical need, driven primarily by the clinical question and the patient population. Assuming such alignment occurs, broader clinical implementation of blood protein biomarkers will still require demonstrated superiority over existing clinical reference standards and favorable health-economic performance. Clinical trials will be crucial in informing public health policy about the benefit of a new blood protein biomarker in terms of diagnostic accuracy, treatment response, or survival. In comparison to existing clinical standards, acceptable cost and gain in benefit-to-cost ratios must be demonstrated.

A simplified illustrative scenario highlights these challenges. In a cardiovascular risk prediction setting, for example, large-scale discovery efforts may identify a multi-protein signature predictive of future cardiovascular events. Translation of such a signature would require development of a targeted panel, demonstration of its measurement reproducibility across laboratories, definition of a clinically actionable decision threshold (e.g., initiation or intensification of preventive therapy), and validation of both individual analytes and the composite score under regulated conditions. Clinical implementation requires prospective cohort studies demonstrating that the panel predicts future cardiovascular events with performance superior to existing clinical risk models and biomarkers. This must be followed by interventional trials showing that its use changes the molecular scores and improves patient outcomes by enabling more effective preventive treatment decisions. This process requires coordinated input from discovery scientists, clinicians, assay developers, regulators, health economists, and health policymakers to ensure that analytical performance, clinical utility, and implementation constraints are aligned early on. Variation between studies of similar patients is certain to emerge in light of the heterogeneity of such patient groups.

Bridging this divide requires a shift in development logic:

- Define the clinical decision and outcome metrics before expanding assay scope.
- Ensure cross-platform reproducibility during discovery rather than post hoc.
- Develop targeted, fit-for-purpose panels optimized for robustness and turnaround time.
- Align validation frameworks with FDA/EMA expectations from early stages.
- Validate clinical utility through prospective clinical trials and re-sampling.
- Establish reimbursement strategies based on demonstrated clinical utility and cost-effectiveness.

The transition from exploratory biomarker candidate profiling to clinical diagnostics will not depend on advertising greater analytical depth, but on disciplined assay design, sample quality requirements, reproducibility across technologies, and pre-defined paths for integration into existing healthcare infrastructure. Discovery success alone does not guarantee clinical viability; translational design must begin at the earliest stages of biomarker assay development.

## Concluding remarks

**Fig. 1:**
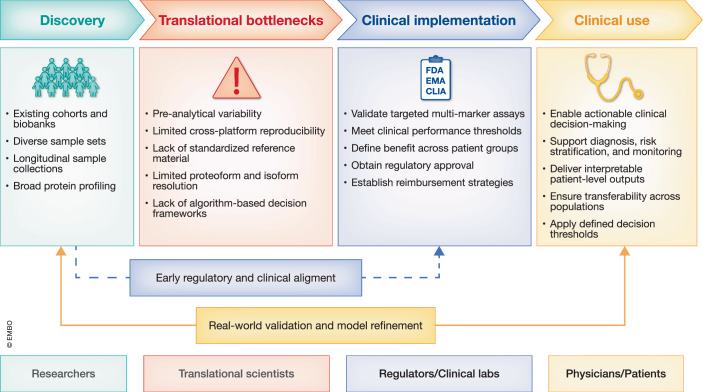
Discovery efforts in population cohorts enable deep profiling of circulating proteins and identification of candidate biomarker signatures. Translation into clinical practice is constrained by key bottlenecks, including limited cross-platform reproducibility, pre-analytical variability, lack of standardized reference materials, incomplete proteoform annotation, and challenges in validating multi-analyte and algorithm-based tests. Clinical implementation requires the development of targeted multiplex assays, the establishment of analytical performance, and regulatory approval under frameworks such as the FDA, EMA, and CLIA, alongside integration into clinical workflows. In clinical use, these assays must deliver actionable outputs that inform defined decision points, such as diagnosis, risk stratification, or treatment selection. A feedback loop from clinical use to earlier stages captures longitudinal monitoring and real-world validation, enabling iterative model refinement under regulatory oversight and supporting continuous improvement of proteomic diagnostics.

Blood-based proteomics has reached a translational inflection point where analytical capability is no longer the primary limitation. Instead, the challenge now lies in converting high-dimensional molecular measurements into reproducible, interpretable, and clinically actionable outputs. The bottlenecks outlined here are interconnected, covering standardization, biological resolution, validation, and regulatory pathways for multi-analyte signatures, and the gap between discovery and implementation. These barriers must be addressed together and in parallel (Fig. 1).

Progress will require a shift from focusing solely on the exploratory hunt for analytical depth toward fit-for-purpose precision assay design anchored in intended clinical use. Standardized workflows, shared reference materials, and a clear and informed definition of the regulated entity are essential to enable reproducibility and regulatory-grade validation. As demonstrated in genomics, clinical utility, not analytical complexity, ultimately determines adoption.

Realizing the clinical potential of proteomics will depend on earlier alignment across technology, discovery, regulatory, and clinical domains, and on embedding new biomarker assays within cost-efficient workflows that support actionable decision-making. If these conditions are met, blood-based proteomics can evolve from a mainly discovery-driven discipline into a reliable cornerstone of clinical care and precision medicine.
